# Supplementary material for: Design and Characterization of pMyc/pMax Peptide-Coupled Gold Nanosystems for Targeting Myc in Prostate Cancer Cell Lines
Source: Nanomaterials (Basel). 2023 Oct 21;13(20):2802. doi: 10.3390/nano13202802 (PMC10609645; doi:10.3390/nano13202802)
Supplement: Supplementary file 1 [file nanomaterials-13-02802-s001.zip › Supplementary tables.pdf]

Table S1. Raw data from ImageJ analysis of Figure S3 of TEM image of pMyc:AuNPs.

	Area	Mean	Min	Max	Angle	Length (nm)
**1**	1943.735	68.621	51.239	95.440	0	292.605
**2**	2109.159	62.647	29.000	103.500	0	321.543
**3**	1985.091	70.849	50.298	111.918	-7.958	301.943
**4**	2315.940	120.010	93.754	147.750	-0.526	350.497
**5**	1819.667	116.475	86.740	196.000	-1.348	273.388
**6**	2481.364	76.629	46.291	121.225	39.867	381.229
**7**	1447.462	97.139	74.913	129.000	65.695	218.744
**8**	2481.364	57.735	30.552	116.000	-62.354	381.134
**9**	1488.818	102.137	83.409	118.500	-65.283	223.004
**10**	1612.887	115.093	87.500	162.000	-64.440	245.933
**11**	1943.735	81.759	46.565	131.000	7.431	298.326
**12**	1530.174	123.259	95.250	156.500	-4.764	232.314
**13**	1654.243	119.655	93.657	157.169	11.889	249.730
**14**	1902.379	128.592	97.462	162.234	16.821	288.888
**15**	1282.038	91.581	44.000	130.000	0.000	192.926
					**Average length (nm)**	283.480
					**SD**	± 57.95

Table S2. Raw data from ImageJ analysis of Figure S3 of TEM image of pMyc:pMax:AuNPs.

	Area	Mean	Min	Max	Angle	Length (nm)
**1**	2274.584	200.638	145.198	254.160	45	350.143
**2**	1488.818	124.461	82.250	157.700	14.036	225.379
**3**	1861.023	164.984	83.000	210.364	-5.839	284.434
**4**	1530.174	148.989	87.000	201.701	-85.972	228.861
**5**	1488.818	119.054	37.575	174.981	15.038	223.073
**6**	1902.379	159.780	89.500	191.000	13.570	287.777
**7**	2150.515	203.300	160.118	230.810	41.820	327.911
**8**	1654.243	175.725	5.000	247.154	-81.254	253.755
**9**	2274.584	192.867	126.407	250.556	-87.879	347.505
**10**	2067.803	130.117	65.000	228.843	8.297	311.947
**11**	1695.599	130.772	5.925	249.200	4.289	257.957
**12**	2688.144	219.341	112.000	255.000	12.724	408.752
**13**	1447.462	135.923	88.000	154.685	-45.000	218.271
**14**	2150.515	166.160	110.997	235.318	40.236	328.541
**15**	1364.750	151.186	98.000	172.750	3.576	206.189
**16**	1985.091	220.556	148.838	255.000	-32.005	303.343
**17**	1199.326	229.863	112.000	255.000	-49.399	177.869
					**Average length (nm)**	278.924
					**SD**	± 63.267

Table S3 Comparative table of NS physical characteristics, peptide concentration, and cytotoxicity in the different cell types.

	Diameter (nm)	Hydrodynamic diameter (nm)	Zeta potential	Peptide concentration (nM)	Highest Cytotoxicity Vero CCL81 (%)	Highest Cytotoxicity LNCAP (%)	Highest Cytotoxicity PC-3 (%)	Highest Cytotoxicity DU145 (%)
**AuNPs**	5.68 ± 0.84	243.03 ± 12.83	-9.05 ± 6.09	0.00	36.32	2.18	0	20.43
**pMyc:AuNPs**	3.28 ± 1.16	264.97 ± 4.39	-5.05 ± 3.42	163.60	36.94	13.27	0	16.4
**pMax:AuNPs**	5.60 ± 1.19	262. 57 ± 23.40	-7.67 ± 5.71	200.86	25.43	0	0	43.01
**pMyc:pMax:AuNPs**	5.78 ± 1.41	225.03 ± 17.65	- 10.09 ± 4.04	223.93	4.11	17.75	7.1	0
