# Supplementary figures and images for: Design and Characterization of pMyc/pMax Peptide-Coupled Gold Nanosystems for Targeting Myc in Prostate Cancer Cell Lines
Source: Nanomaterials (Basel). 2023 Oct 21;13(20):2802. doi: 10.3390/nano13202802 (PMC10609645; doi:10.3390/nano13202802)

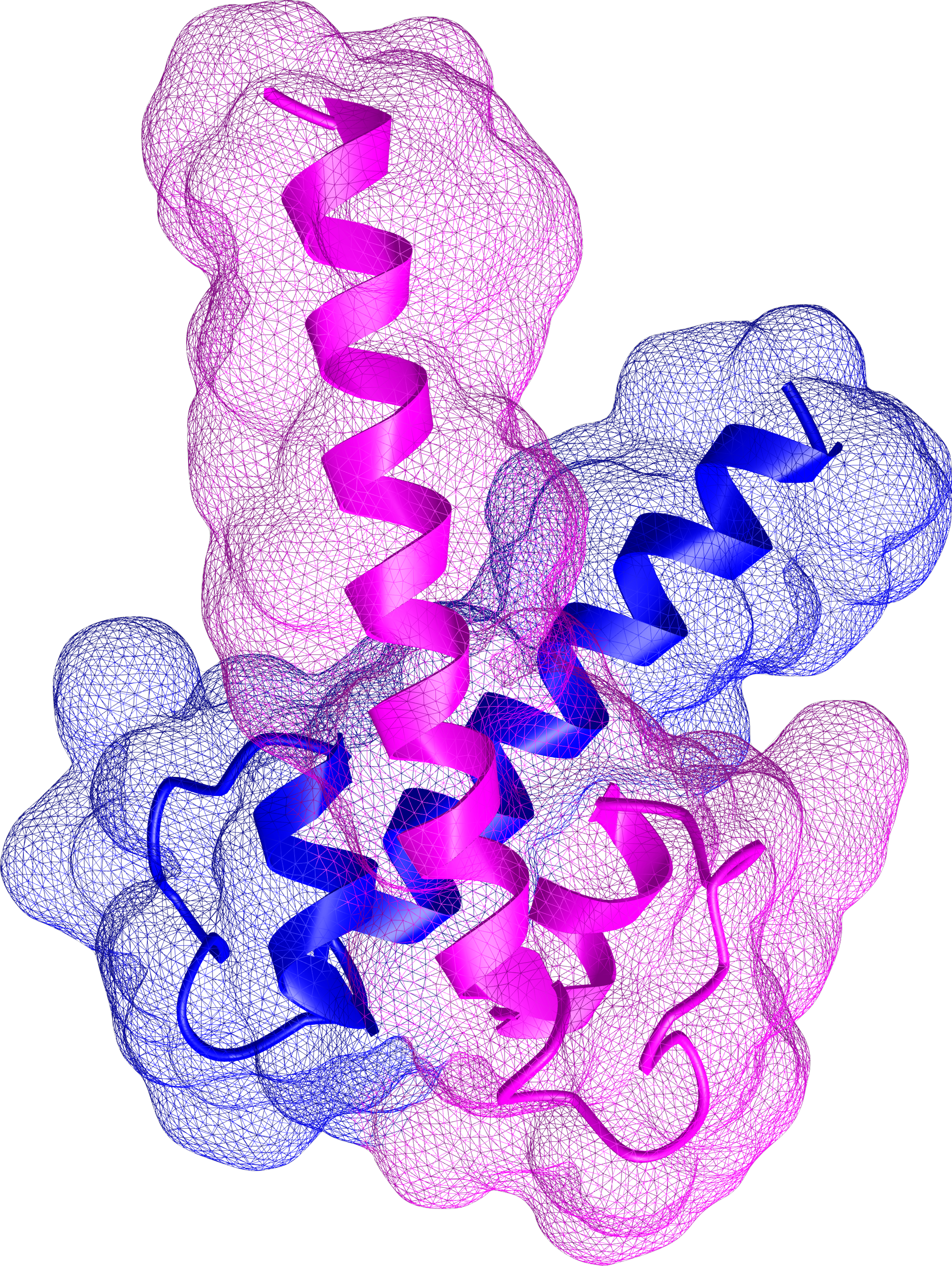

Supplement: Supplementary file 1 [file nanomaterials-13-02802-s001.zip › Figure S1 pMyc and pMax modelling.tiff]

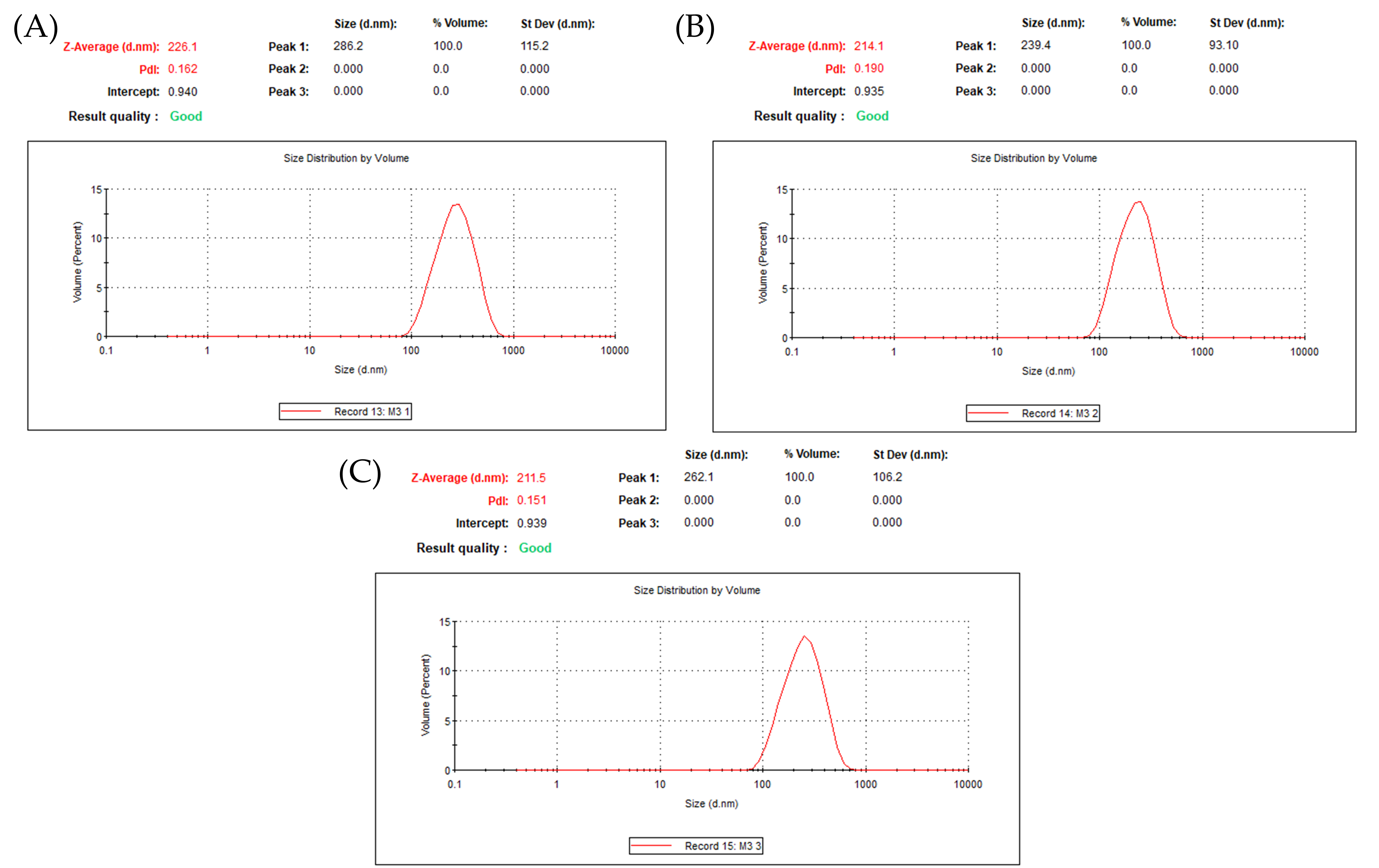

Supplement: Supplementary file 1 [file nanomaterials-13-02802-s001.zip › Figure S10. pMaxAuNPs HD DLS data.tif]

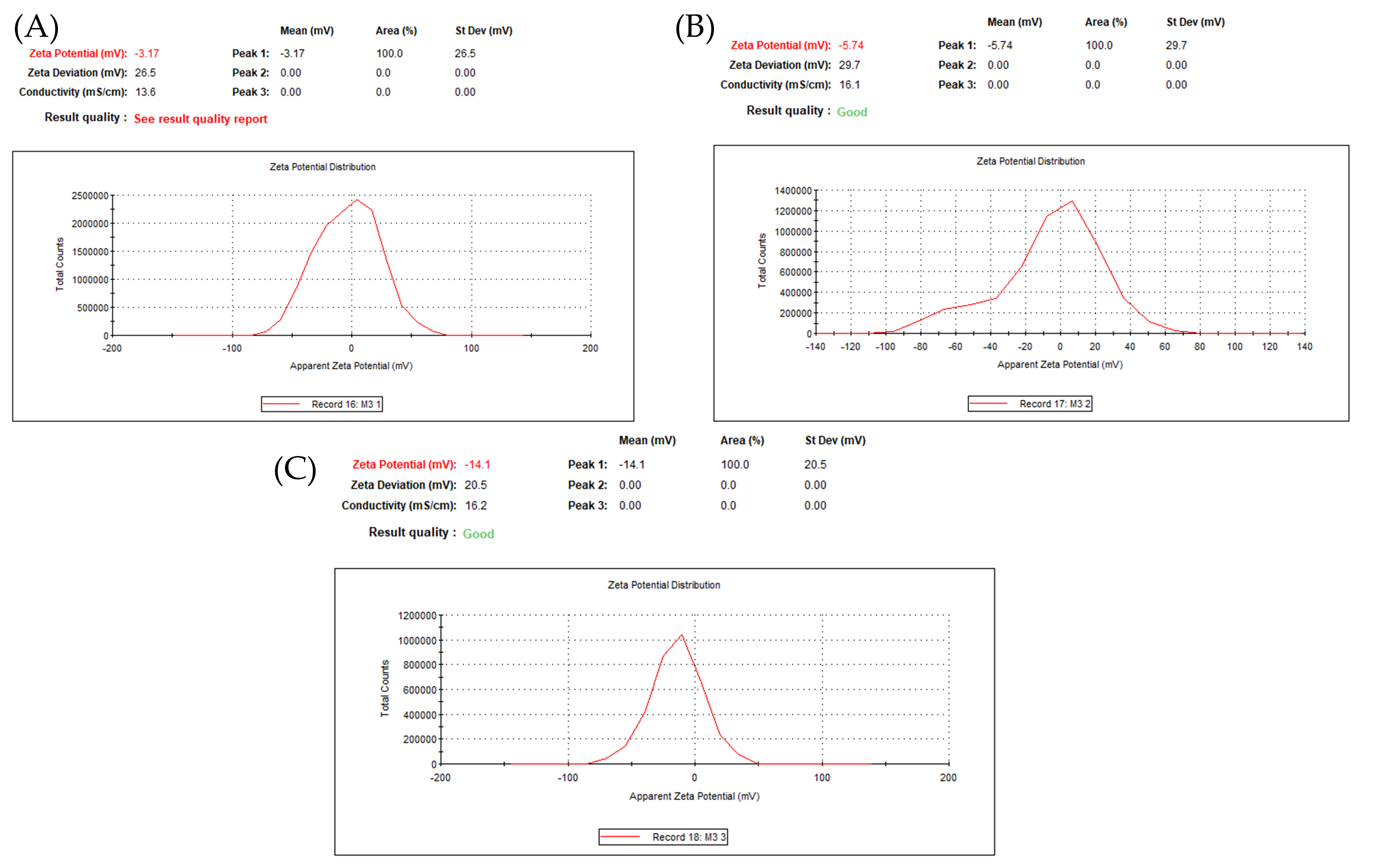

Supplement: Supplementary file 1 [file nanomaterials-13-02802-s001.zip › Figure S11. pMaxAuNPs zeta potential DLS data.tif]

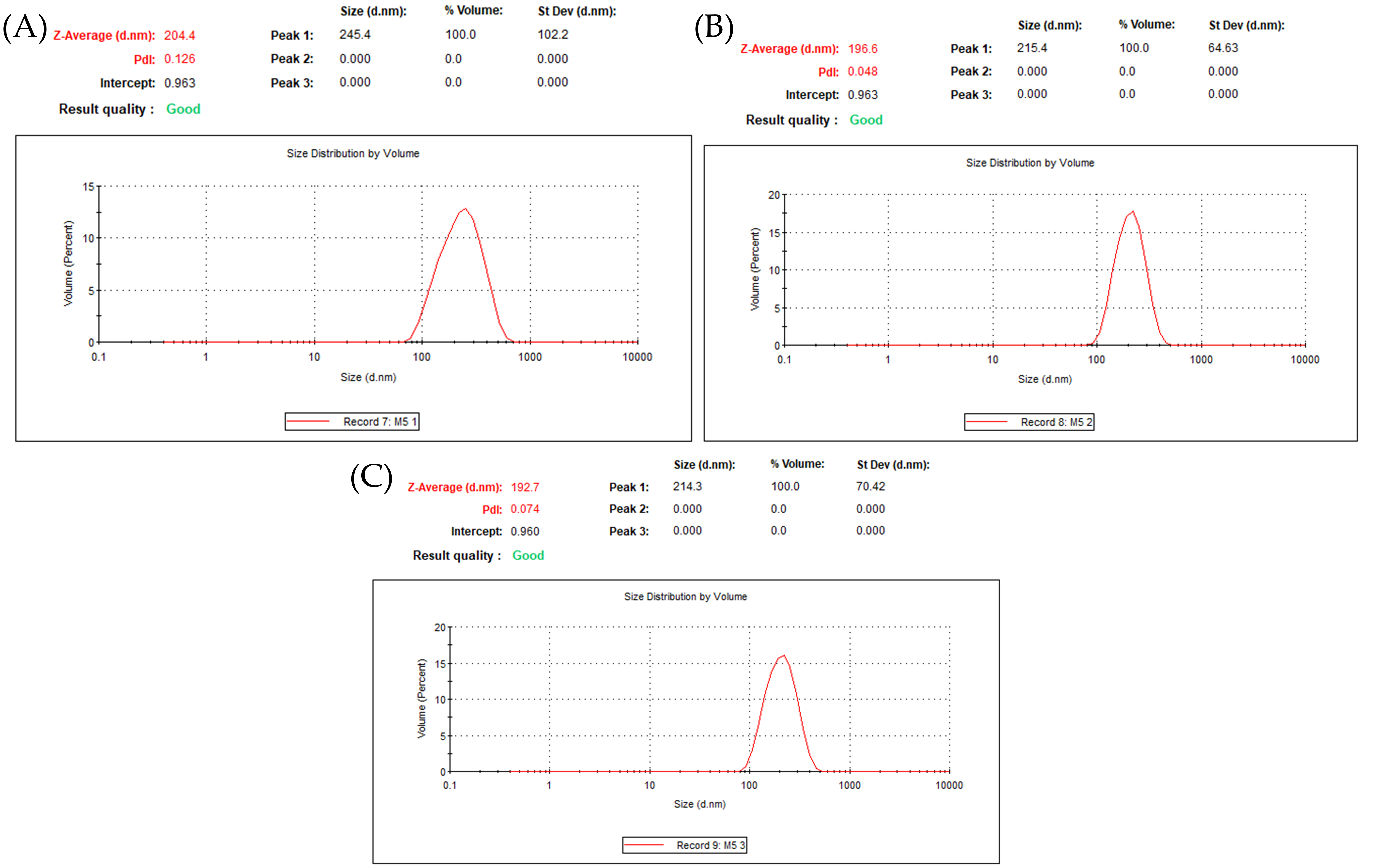

Supplement: Supplementary file 1 [file nanomaterials-13-02802-s001.zip › Figure S12 pMycpMaxAuNPs HD DLS data.tif]

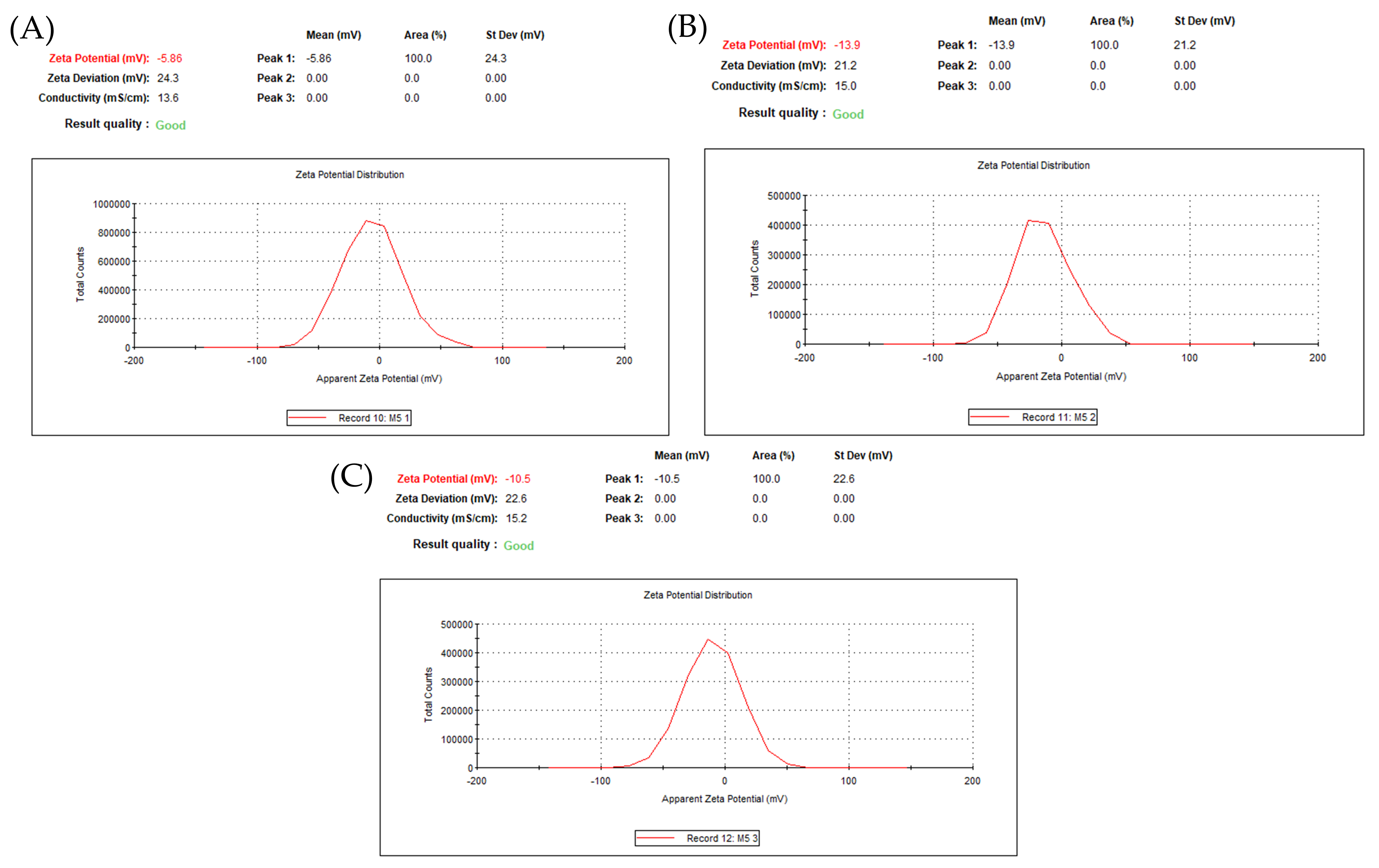

Supplement: Supplementary file 1 [file nanomaterials-13-02802-s001.zip › Figure S13. pMycpMaxAuNPs zeta potential DLS data.tif]

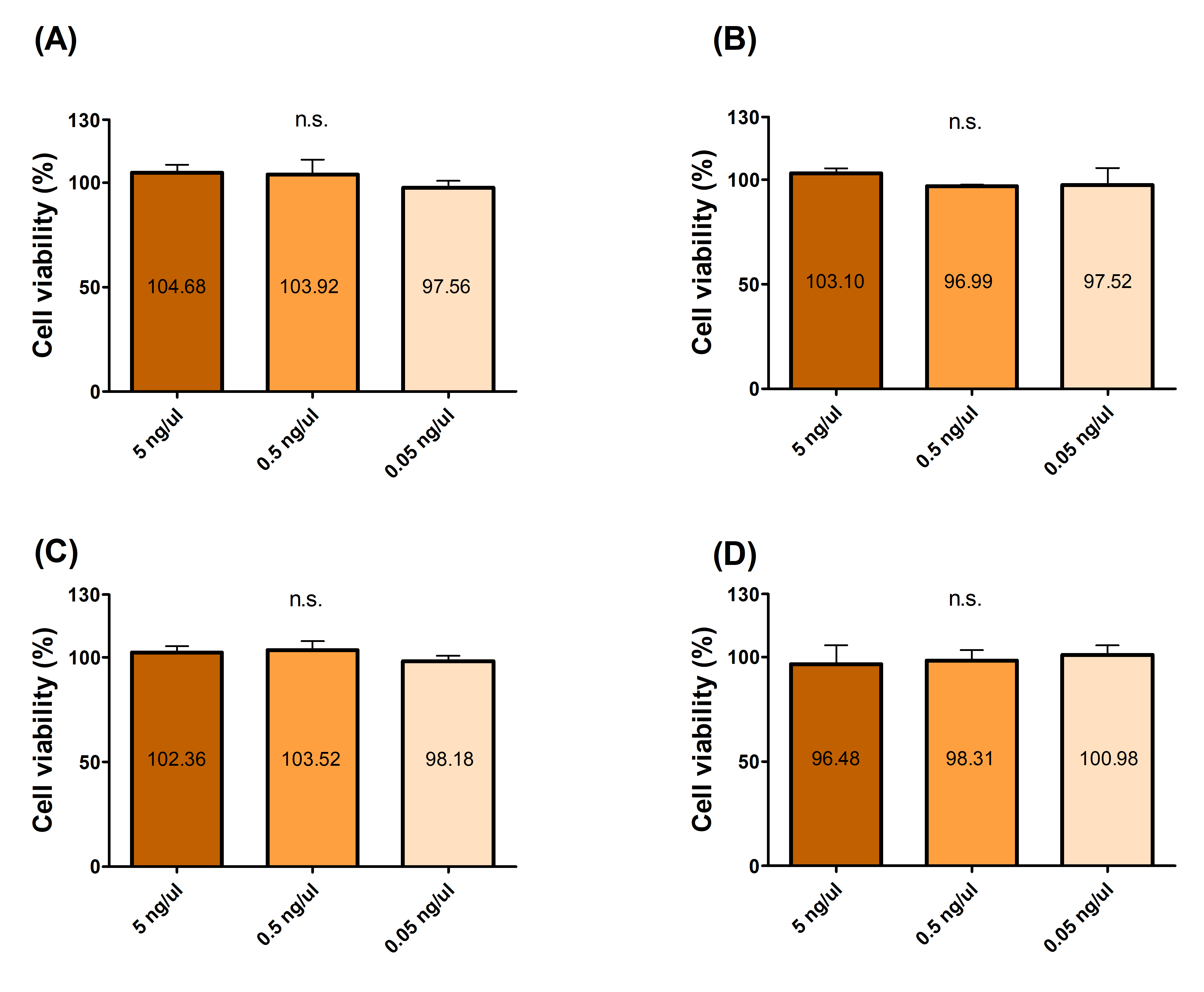

Supplement: Supplementary file 1 [file nanomaterials-13-02802-s001.zip › Figure S2 Cell viability only peptides.tif]

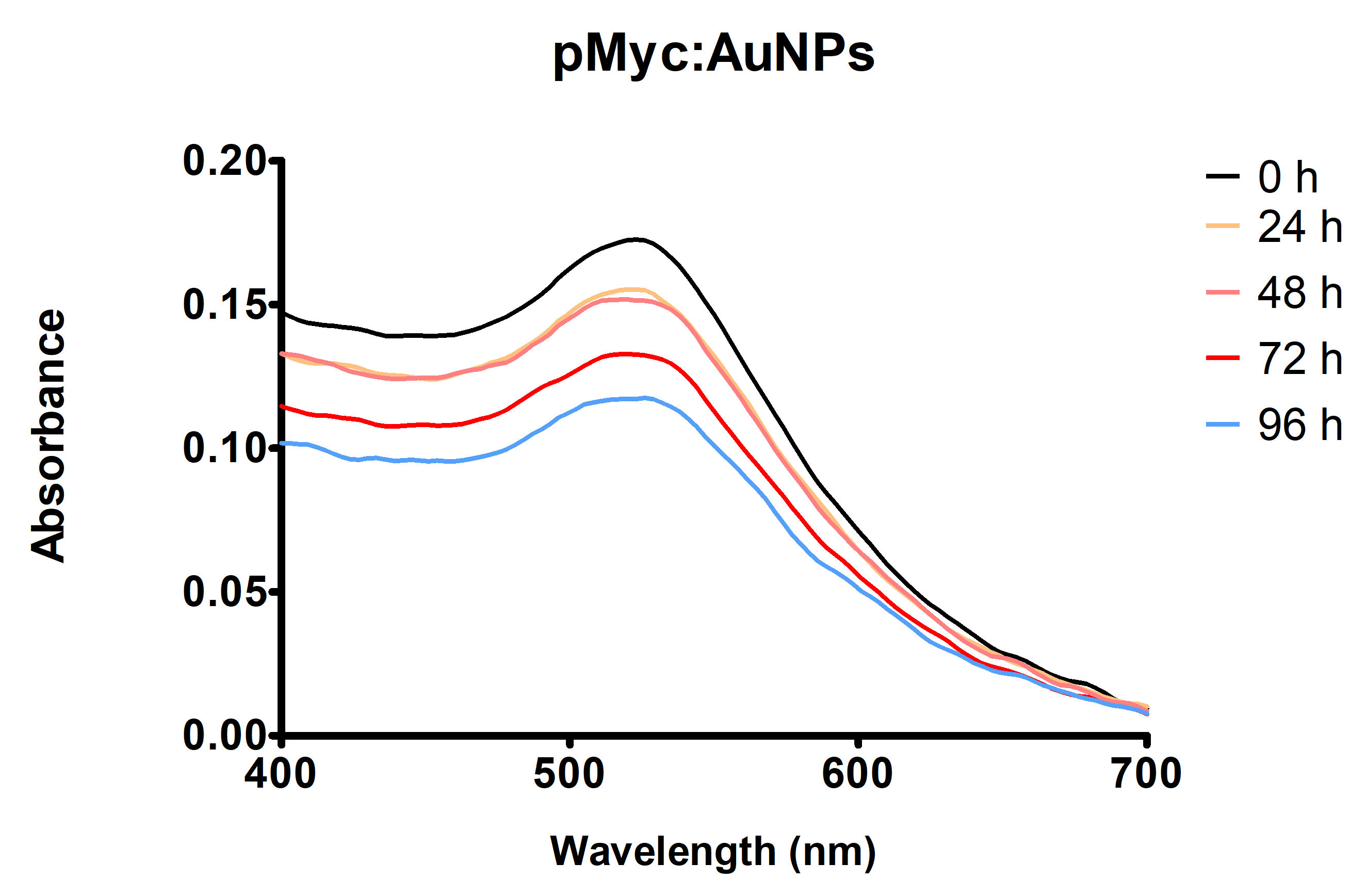

Supplement: Supplementary file 1 [file nanomaterials-13-02802-s001.zip › Figure S3. UV-Vis Spectra pMyc stability.tif]

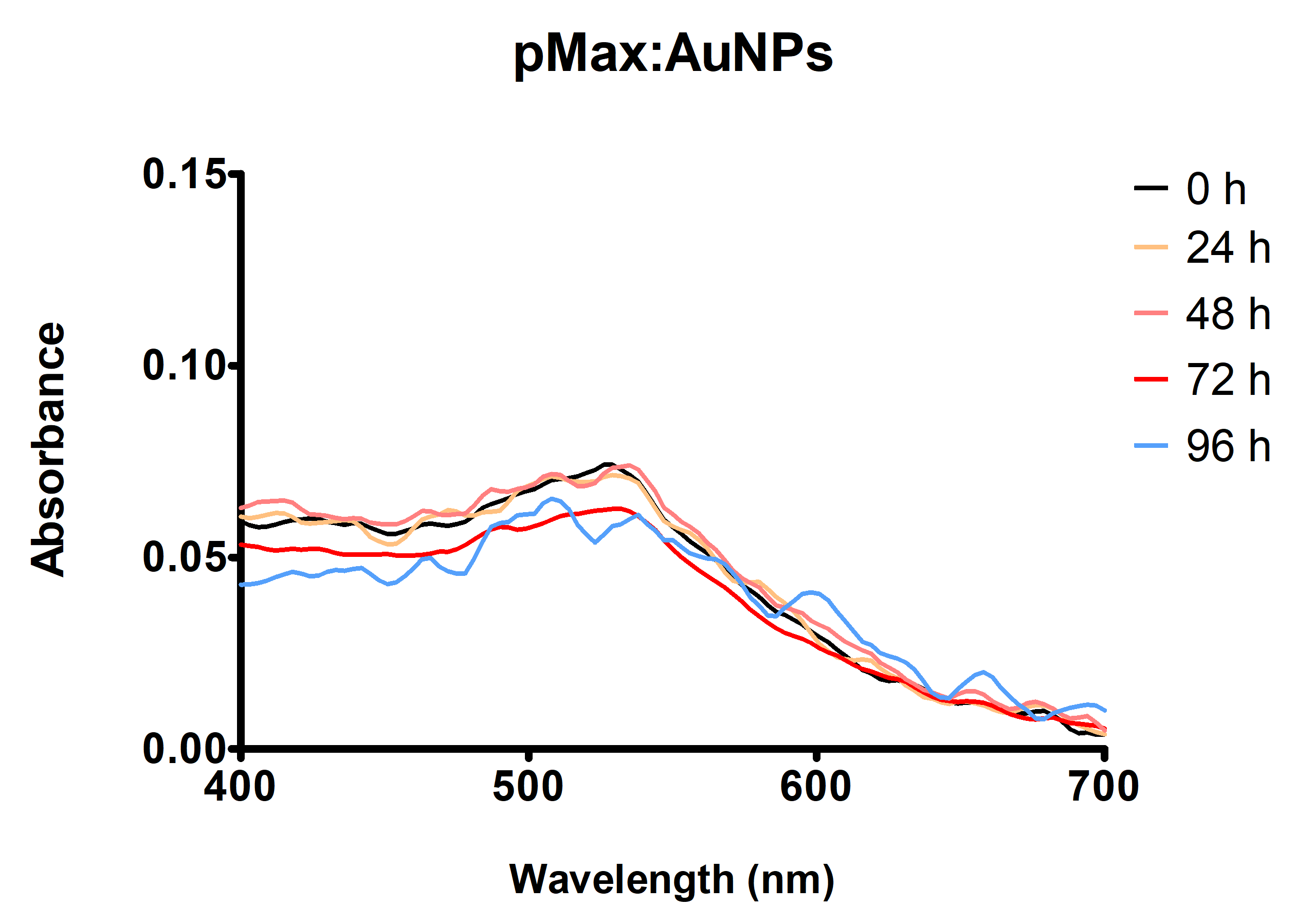

Supplement: Supplementary file 1 [file nanomaterials-13-02802-s001.zip › Figure S4. UV-Vis spectra pMax stability.tif]

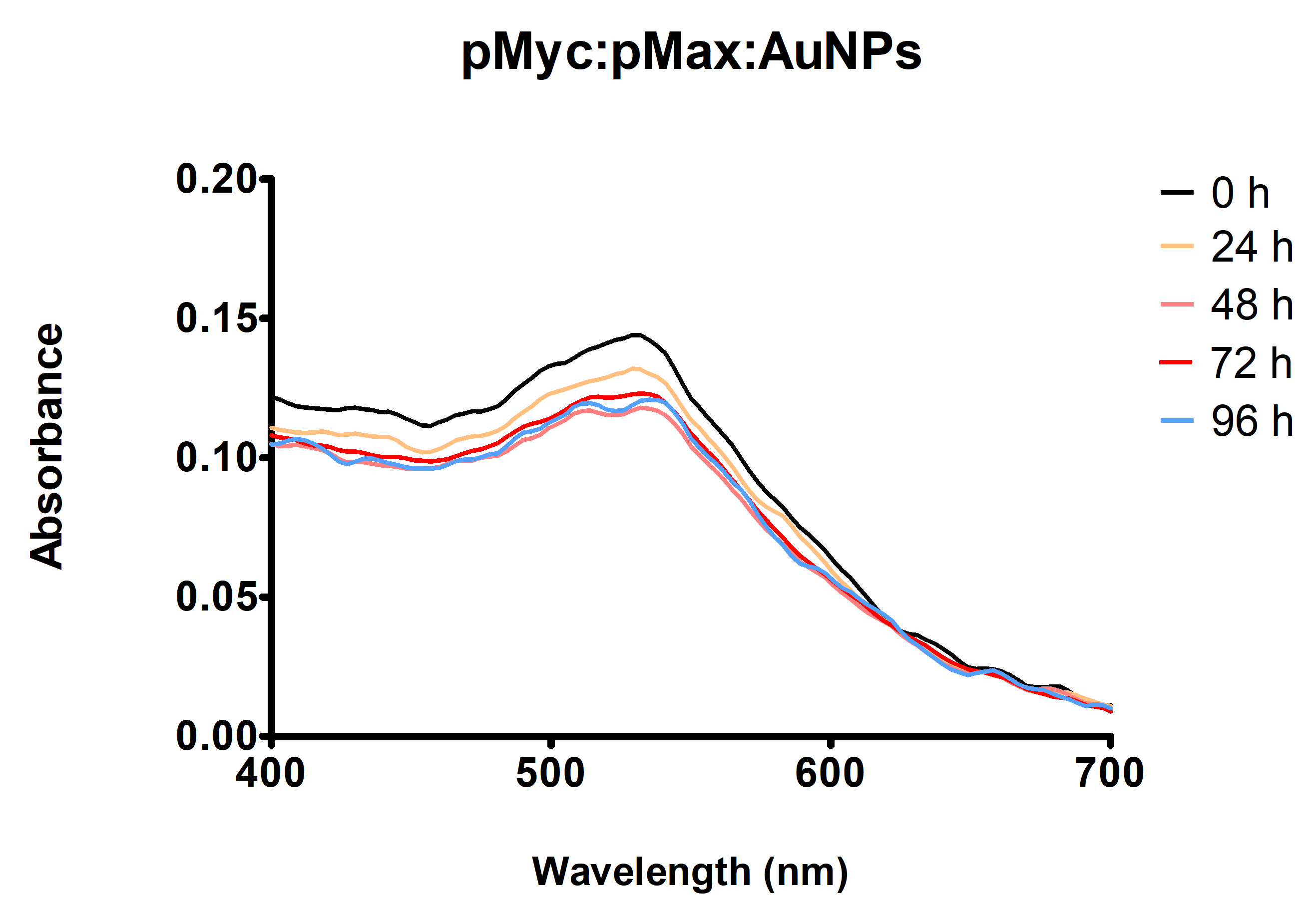

Supplement: Supplementary file 1 [file nanomaterials-13-02802-s001.zip › Figure S5. UV-Vis spectra pMycpMax stability.tif]

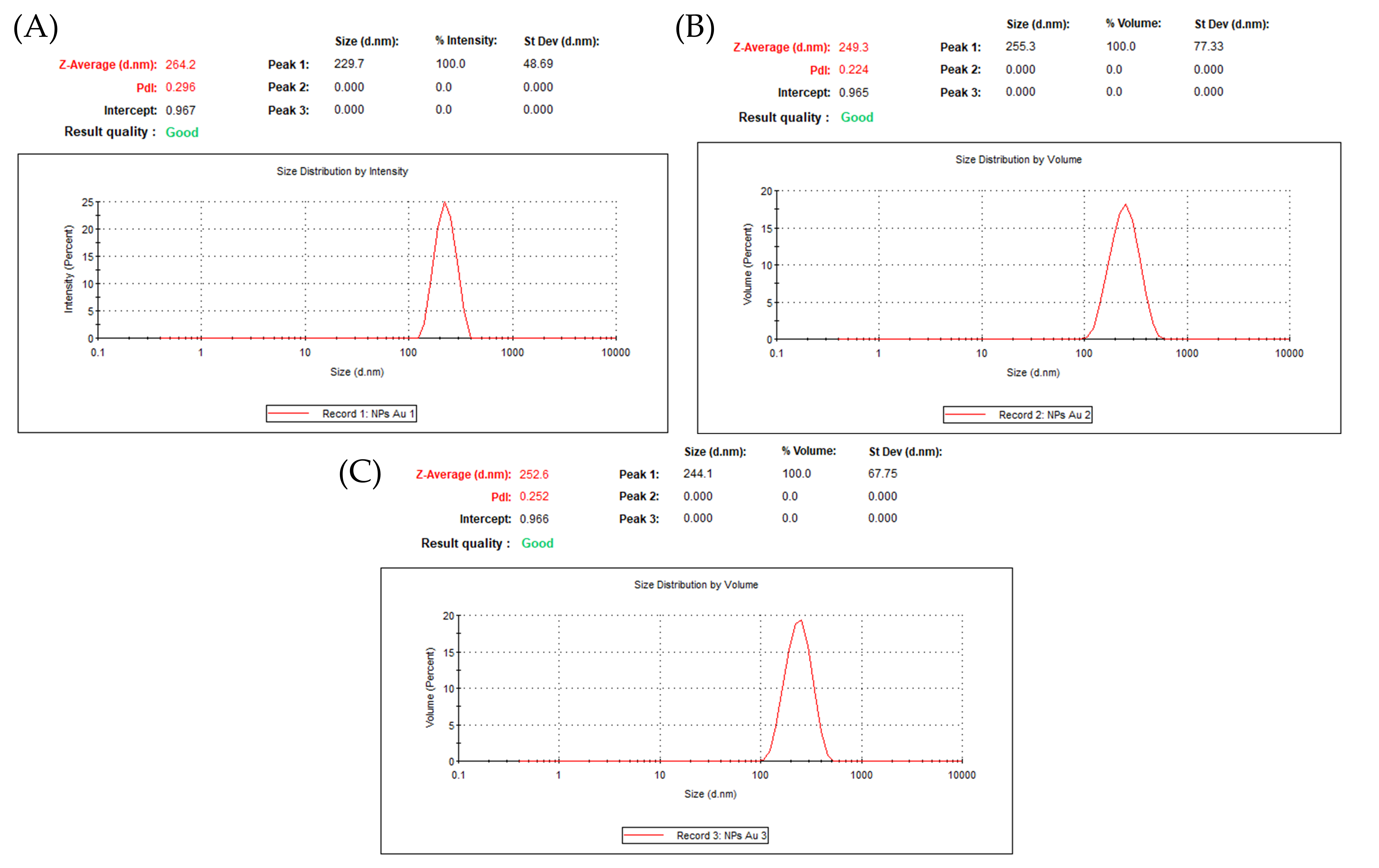

Supplement: Supplementary file 1 [file nanomaterials-13-02802-s001.zip › Figure S6. AuNPs HD DLS data.tif]

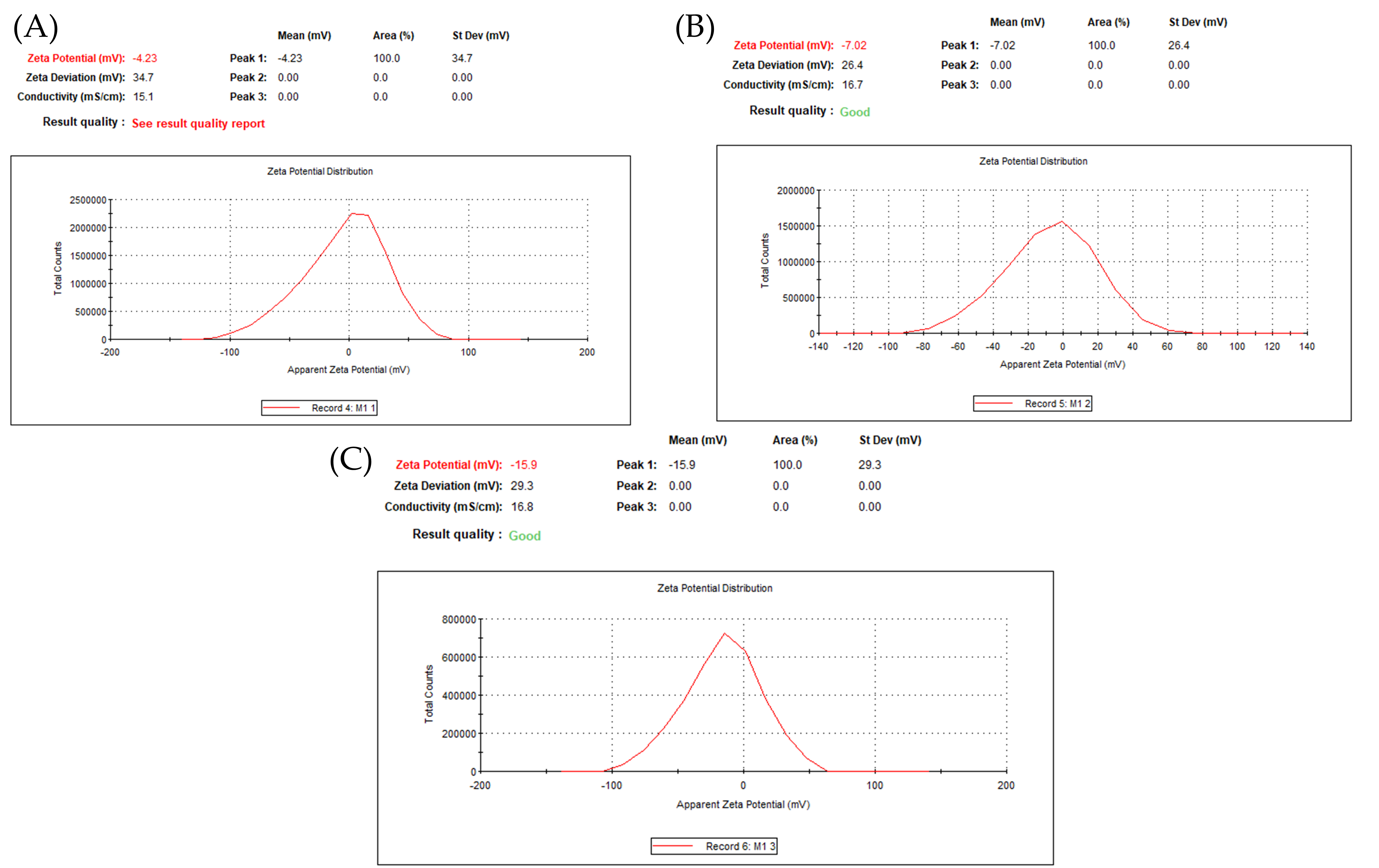

Supplement: Supplementary file 1 [file nanomaterials-13-02802-s001.zip › Figure S7. AuNPs zeta potential DLS data.tif]

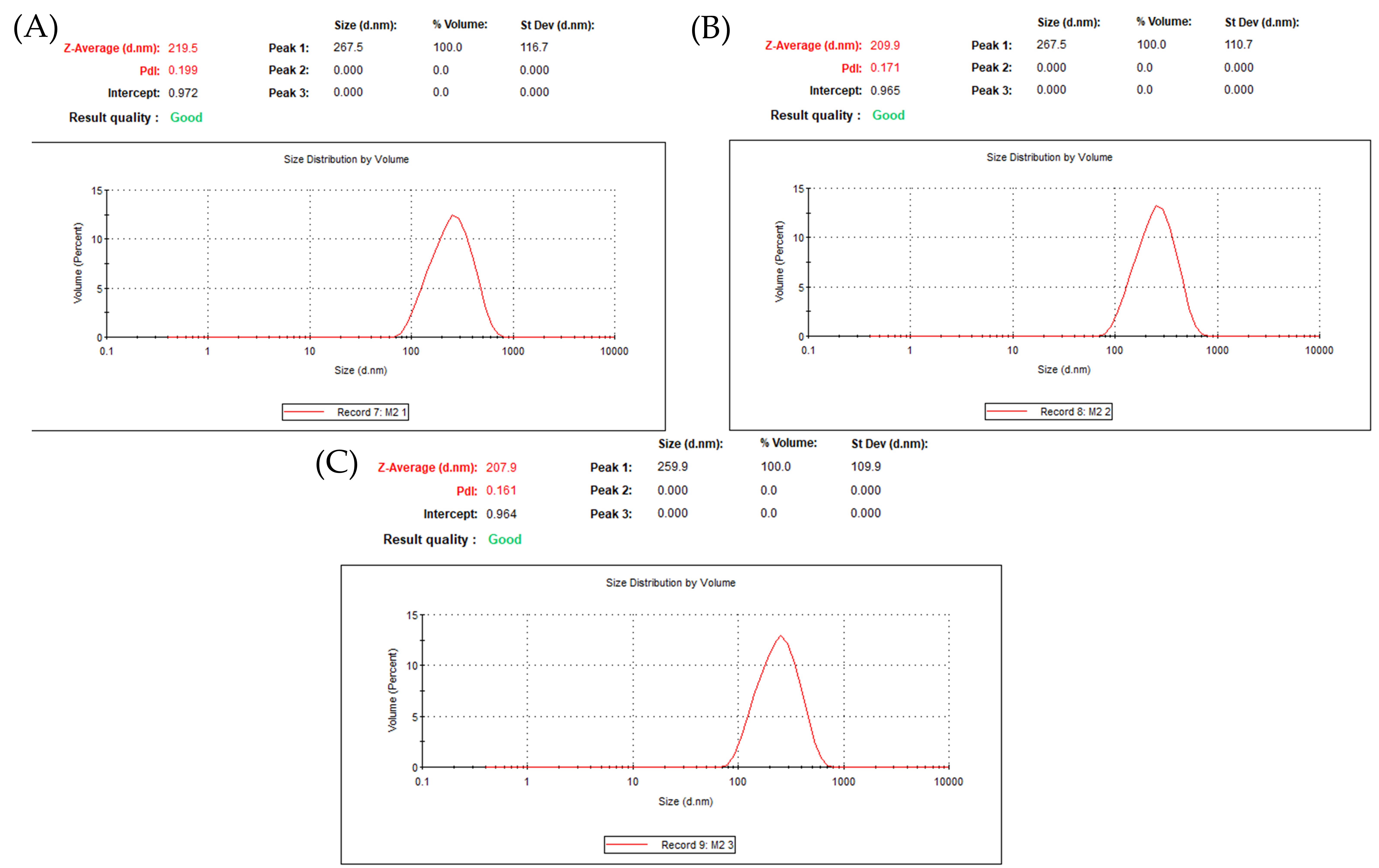

Supplement: Supplementary file 1 [file nanomaterials-13-02802-s001.zip › Figure S8. pMycAuNPs HD DLS data.tif]

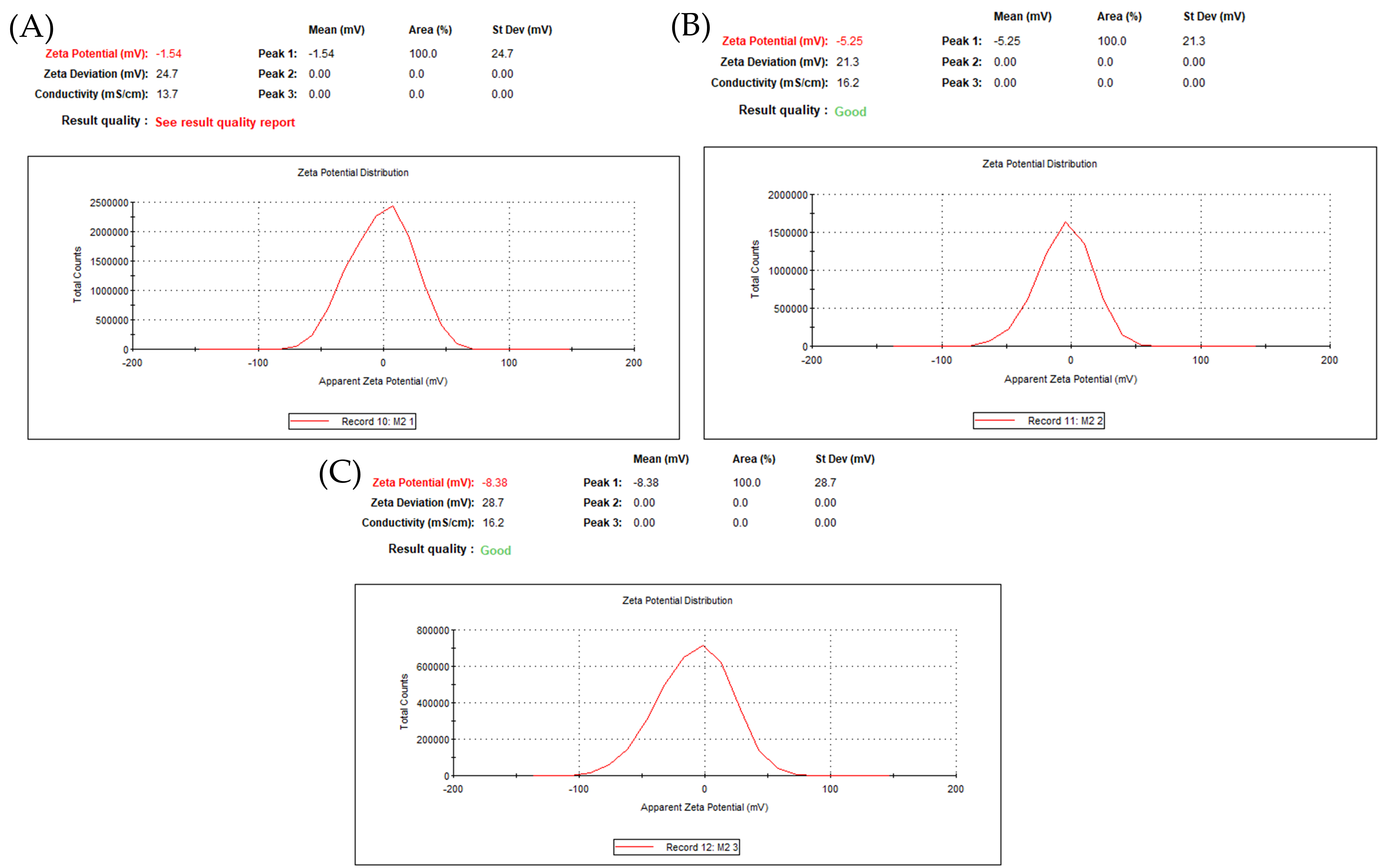

Supplement: Supplementary file 1 [file nanomaterials-13-02802-s001.zip › Figure S9. pMycAuNPs Zeta potential DLS data.tif]
